# A qualitative study of patients and healthcare workers’ experiences and perceptions to inform a better understanding of gaps in care for pre-discharged tuberculosis patients in Cape Town, South Africa

**DOI:** 10.1186/s12913-022-07540-2

**Published:** 2022-01-29

**Authors:** Idriss I. Kallon, Christopher J. Colvin, Zara Trafford

**Affiliations:** 1 Division of Social and Behavioural Sciences, School of Public Health and Family Medicine, University of Cape Town, Cape Town, South Africa; 2 Centre for Evidence-based Health Care, Division of Epidemiology and Biostatistics, Department of Global Health, Stellenbosch University, Cape Town, South Africa; 3 Department of Public Health Sciences, University of Virginia, Virginia, USA; 4 Department of Epidemiology, Brown University, Providence, USA; 5 Psychology Department, Stellenbosch University, Stellenbosch, South Africa

**Keywords:** Patient-centred care, Continuity of care, Tuberculosis, Drug-resistant tuberculosis, South Africa

## Abstract

**Background:**

Many people diagnosed with *Mycobacterium tuberculosis* (TB) in tertiary and district hospitals in South Africa do not arrive at their primary care clinic for continued care after they are discharged from the hospital. This loss to follow up is a major, ongoing problem for public health in South Africa, and contributes to drug-resistant TB strains. The objective of this paper was to explore patients’ experiences and perceptions of diagnosis and treatment before their discharge from hospital. We use a framework known as patient-centred care to illustrate how these patient narratives point to lapses in these principles within the hospital system, and to show how such lapses may contribute to loss to follow up and inconsistent TB care.

**Methods:**

We employed a qualitative study using semi-structured interviews to investigate patient and healthcare workers’ experiences and perceptions of TB care in two Western Cape hospitals. We purposefully sampled 17 patients, 10 healthcare workers, and two key informant policy makers, all of whom had relevant experiences and insights. Data collection was done between October 2015 and February 2017. Data were analysed using Miles and Huberman’s qualitative analysis framework.

**Results:**

Hospitals did not achieve patient-centred care. Newly diagnosed patients were provided with inadequate TB education, diseased-focused approaches were favoured over patient-focused approaches, and there was limited engagement with patients to understand their needs and feelings during the critical period between diagnosis and discharge. Consequently, some patients felt anxious prior to their discharge from hospital. Coupled with their overwhelming socio-economic barriers and complex family situations, some patients felt hopeless and powerless as they prepared for discharge. Finally, there was a lack of patient-provider partnership due to problems including healthcare workers’ time constraints and heavy workloads, which detracted from a focus on patients’ needs and feelings.

**Conclusions:**

Improving the three intersecting elements of patient-centred care (health education, engaging with patients’ needs and feelings, and shared decision-making) has the potential to positively influence patients’ continuity of care for TB in South Africa. It would be helpful to also proactively address how patients plan to stay connected to care, on treatment, and supported, in light of their family situation or socio-economic circumstances. Detailed and unique pre-discharge counselling for each patient may be valuable in this regard.

**Supplementary Information:**

The online version contains supplementary material available at 10.1186/s12913-022-07540-2.

## Background

Tuberculosis (TB) is the main cause of mortality in the country [1, 2]. Although effective TB treatment exists, there are ongoing and persistent problems with infection control as well as access and adherence to treatment. Poor continuity of and retention in care are of particular concern. For example, a 2005 study conducted in a hospital in one South African province (Gauteng) found that 50% of the 407 patients diagnosed in the hospital had only reported to a primary health care (PHC) clinic 2 weeks after their TB medication ran out [3]. In 2018, another study conducted in the Western Cape reported that of the 788 people hospitalised for TB in one tertiary hospital, only 284 (36%) had continued their TB treatment at a local clinic after discharge [4]. This loss to follow up points to the organisation of the South African health system that are worth exploring to understand the context of this study.

Following a formal restructuring of the health services and a re-orientation to primary health care principles in the 1990s, the South African health system is now, in theory, decentralised and tiered. Individuals are supposed to present at their local PHC when they fall ill, receive a diagnosis if relevant, and then obtain whatever treatment is available at this primary care facility. If they require specialised or additional care, they should be referred “up” the tiered system to a secondary (“district”) or tertiary hospital [5]. Since 1996, South Africa has applied the World Health Organisation’s (WHO) treatment protocols for TB, known as directly observed therapy, short course (“DOTS”), which entails a patient taking their daily treatment while observed by a nurse, family member, friend, or community health worker [6]. According to the DOTS protocol and the tiered South African health system, ideally, people with TB should be diagnosed and have their condition managed at their local PHC [7]. However, many patients are actually diagnosed with TB at either tertiary or district hospitals. Following an in-hospital diagnosis and initiation of treatment, they are then discharged and referred “down” to local clinics for continuity of care (CoC) [7]. Many people diagnosed with TB in tertiary and district hospitals, however, do not present at their clinic for continued care after they are discharged from the hospital [3, 8, 9]. This loss to follow up is a major, ongoing problem for public health in South Africa, and contributes to the proliferation of drug-resistant TB (DR-TB) strains [4].

CoC has been conceptualised in two main ways. One refers to patients receiving services from the same physician over time, but this is less relevant in the South African public sector as it is uncommon for patients to regularly see the same physician due to rotation systems and resource shortages [10]. The term CoC can also be used to refer to the effective transfer of patients’ information from one health centre to another, and ensuring consistency in patients’ interactions with different health practitioners [11, 12]. This study uses the latter conceptualisation because it focuses on the participants’ treatment journey, which includes the transfer of information from the hospital to the clinic. Research shows that proper notification, referral and followup between the clinic and the hospital is the foundation of good CoC [9]. Lack of proper communication between different tiers of the health system (e.g. between a hospital and a clinic) can negatively impact on treatment continuity and is economically expensive for the system [6]. In a 2018 study in the Western Cape, poor CoC was found to be associated with fragmented data and information systems throughout the health system, not being an adult (i.e. not being personally responsible for one’s own care and being monitored by an older person), living in a rural residence, not having been diagnosed with TB at a hospital, and not having received TB medication on discharge [4]. Further factors that can negatively affect CoC include clinical hierarchies among staff, insufficient staff to engage in TB health education, and poor discharge planning on the part of health staff [9].

It is important to gain a better understanding of inpatient clinical management and the discharge phase of treatment from the perspective of the people who experience these processes as recipients of care. However, most studies to date have drawn heavily on healthcare workers’ (HCWs) perspectives about treatment and CoC. There is little qualitative evidence about patients’ experiences of and perspectives on treatment during hospitalisation. More evidence about patient experiences from their own perspective would help us understand why some people do not attend the PHCs they have been referred to for continued TB care, and can also inform health service interventions to address the problem. This also aligns well with the patient-centred care (PCC) approach, which emphasises the importance of patients participating in their own care, the relationshipbetween the patients and their healthcare provider and the existing context where the care is delivered [11]. PCC is the explanatory framework used in this paper and is described further below.

This paper presents findings from a qualitative study which investigated patients’ personal experiences of being diagnosed with and treated for TB in two Western Cape hospitals. Patient data is complemented with findings from interviews with HCWs who managed inpatient clinical care and discharge of the same patients. We also interviewed policy makers who were involved in policy around hospital care for patients in the Western Cape. The main objective of this paper is to contribute to our understanding of patients’ experiences and perceptions of TB diagnosis and treatment before their discharge from hospital. We adopt a framework known as PCC to describe lapses in these principles within the hospital system, and show how these factors may contribute to loss to follow up and lack of CoC.

## Patient-centred care model

**Fig. 1:**
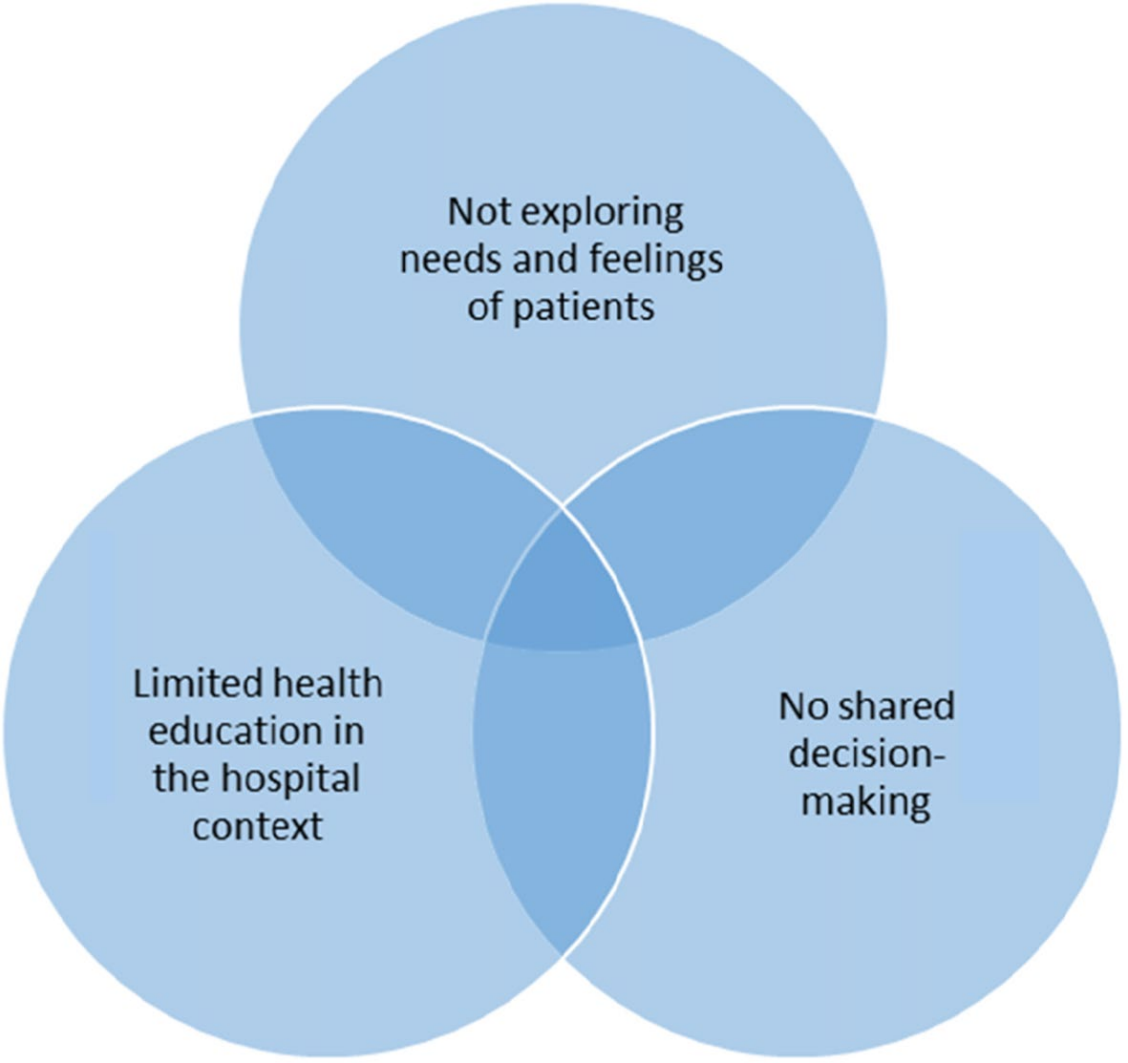
Patient-centred care model explored in this study

The PCC model is a widely used framework for understanding and assessing the quality of the service and care provided to patients in health settings [11–13]. The concept of PCC has evolved from more than 20 years of usage in medicine, nursing, and health policy but its uses have been diverse, and its meaning is not universally agreed upon [11]. A narrative review and synthesis of literature from all of these disciplines was conducted in 2012. The review identified the following core domains which can be analysed to assess if care is adequately patient-centred: 1) the nature of “patients’ participation and involvement in care” (Shared decision-making) 2) the “relationship between the patient and the health professional” (according to patients’ needs and feelings), and 3) the “context where the care is delivered” (including health education in hospitals) [11] (see Fig. 1). The authors conclude that for adequate PCC, patients should be involved in all processes associated with the care that they receive, that each patient should be regarded as a unique person with needs, expectations and fears that should not be generalised, and that patients should receive respectful treatment by health care workers. Further, the context in which care is provided (clinical spaces and health facilities) should be comfortable and hopeful, or patients may feel alienated. Similar themes of PCC are well-supported by South African laws and principles. For example, the South African Constitution states that patients have the right to care that is provided with respect and that incorporates consideration of their needs and values [13]. The *Batho Pele* (“People First”) initiative, which is supposed to guide the provision of service delivery in South Africa, states that patients should have a choice about what “services are being offered to them and should have access to education that explains the services that are rolled out to them…” [13].

## Methods

### Research design

The data in this article are derived from the first author’s doctoral field research, conducted between October 2015 and February 2017 in Cape Town, South Africa. We conducted a qualitative study and data were gathered through in-depth semi-structured interviews. A group of patients were interviewed while at the hospital where they were diagnosed. HCWs and policy makers were also interviewed either regarding the services they provide to patients diagnosed with TB and/or knowledge of discharge planning at the hospital.

### Study setting

The study recruited participants from two hospitals, “Hospital 1” and “Hospital 2”. Hospital 1 is a tertiary, academic hospital located in an urban suburb in Cape Town, in South Africa’s Western Cape province. Hospital 2 is a district hospital located in one of the “townships” in South Africa, also in the Western Cape. Townships are communities in South Africa that were formed because of segregationist “separate development” policies (*apartheid*), which kept Black/African populations away from their White counterparts in South Africa [14, 15]. These apartheid policies were abolished in the late 1980s prior to the country’s first democratic elections in 1994. Townships are generally socio-economically poor areas, and generally situated far from urban centres, service providers, and sources of income [16]. Housing is often overcrowded and sanitation provisions are insufficient. These circumstances contribute to the rapid spread of infectious diseases like TB. Hospital 1 and Hospital 2 were chosen specifically because they regularly treated and referred people newly diagnosed with TB to PHCs to continue their treatment [8, 9]. Internal Medicine wards were used for data collection because they had a high incidence of TB. In Hospital 1, between 2008 and 2010, the Internal Medicine speciality accounted for 24% of TB cases, followed by Paediatrics (22%) and ICU (22%). The incidence in other specialities was 10% or less. Similarly, by 2017, in Hospital 2, the Medical, Paediatrics and Emergency wards accounted for more than 60% of the TB cases.

### Recruitment and sampling

Working relationships with nurses and doctors helped the first author obtain access to relevant personnel, including policy makers in the Western Cape Department of Health (WCDoH) and patients in the relevant ward. Participants were purposefully sampled and included “patients” (people diagnosed with TB at the hospital and the primary participants in this study), health care workers, and local policy makers who were recruited from the WCDoH. Participants were approached if they were newly diagnosed and an adult (18 or older). All patients who were approached agreed to participate in the study. HCWs were included if they had treated one or more of the participating patients. All HCWs approached also agreed to participate in the study. Eligibility criteria for policy makerswere: being employed by the WCDoH, having been involved with drafting or managing TB policies, having worked in the hospital, and having had knowledge about the care that is provided for patients in hospitals prior to discharge to clinics. The two policy makers that fit the recruitment inclusion criteria were secured through the WCDoH. All participants, including policy makers were initially approached via email or face-to-face interactions in the hospitals, their offices, or through phone calls. Once they had expressed an interest in participation, each participant was given an informed consent form, information sheet and contact details for the researcher. Each participant verbally agreed to be part of the study and signed the informed consent form.

### Data collection

Semi-structured interview guides were used with patients. The patient interview guide included reasons patients were admitted, the type of TB they had been diagnosed with, their knowledge of TB and the hospital discharge process, and the role they played in the discharge process. The topics also included the patient’s living conditions and the support they received at home. Interviews were conducted in hospital and ranged from 30 min to an hour, depending on the patient’s physical state and desire to engage. Interviews were as private as possible in the context of a hospital ward. Each interview was audiotaped after securing permission from each participant. These audio recordings were complemented by field notes. Patient interviews were led by the first author, who was assisted by a research assistant fluent in the local languages spoken by the patients. Although all patients could communicate well in English, patients were encouraged to speak in their first language. All the interview questions were written in English but were translated and explained in the local languages of the participants by the research assistant (a research assistant who had training and experience in dealing with patients on HIV/AIDS and TB treatment was also part of the study as an interpreter). Translation of manuscripts was provided for but not needed, because all patients could communicate well in English.

Interviews with HCWs and policy makers were also guided by a semi-structured one-on-one interview conducted by the first author. Topics included their knowledge about the treatment and discharge-planning of patients, and their perceptions of patients’ preparedness for continuing their treatment on discharge from the hospitals. HCWs and policy makers who had consented were interviewed in quiet spaces in their offices in the hospitals and clinics.

### Trustworthiness

The researchers maintained reflexivity during the data collection and analytical phases of the study. This included being explicit about any preconceived ideas about the research and any knowledge of potential bias [17]. Even though we were knowledgeable about TB treatment and some of the challenges that patients faced, the participants were assured that the researchers were not involved in healthcare provision and discharge planning. As the study could inform improved health practices and outcomes and might have included descriptions of unsatisfactory encounters with the health system, participants were encouraged to be honest in their responses. We used the 32-item COREQ checklist to report the research team and reflexivity, study design, analysis and findings (SI.COREQ checklist) [18]. The analytical process was verified by the second and third author to maintain consistency and explore potentials for transferability of study findings.

### Data analysis

Miles and Huberman’s qualitative data analysis framework [19] was used. This approach highlights the interactive nature of thorough qualitative research and facilitates the inductive identification of patterns and themes. There are three main components to this approach. These are data reduction, data display, and drawing and verifying conclusions. These modifications give “direction or “focus” to the research [19]. Using themes selected by the research team, certain concepts are selected for focus, leading to data reduction before the actual analysis starts. The research team can then categorise data that is relevant to the topic and arrive at themes, which are then verified by two researchers. The first author did initial coding, which was verified by the second and third authors. The second and third authors discussed the themes in detail with the first author, who also provided indicative excerpts of the data as evidence for each theme and sub-theme. These themes were refined further after this verification process (SI. Steps of analytical method). Use of the QSR International NVivo11 software package facilitated systematic data analysis, electronic coding, and clear organisation of the data [20], as well as preventing data loss. After an initial inductive analysis of the data was conducted, we used the above-mentioned PCC model and its three key domains—shared decision-making, the patient-provider relationship, and the health care context—as a broad conceptual framework with which to organize the key findings and write up the final analysis.

## Results

All participants consented to being interviewed. Written and verbal informed consent was obtained from each participant. The final sample that provided data saturation were twenty-nine (29) participants: seventeen (17) were patients who had been diagnosed with TB at the research site hospitals, ten (10) were HCWs (six nurses, two doctors, and two social workers), and two (2) were local policy makers.

### Patient participant demographics

Seventeen (17) patients participated in the study ranging in ages from 18 to 50 years old. Thirteen (13) of these people were diagnosed at Hospital 1 and four (4) were diagnosed at Hospital 2. Eleven (11) were female and six (6) were male. Two of the 17 participants were diagnosed with Multidrug-resistant TB (MDR-TB). Seven (7) of the participants had HIV as a comorbidity. The mean age of the group of patients was 35 years. Only four (4) patients had finished secondary school and only one had attained a tertiary education. Twelve (12) spoke IsiXhosa as their first language and five (5) spoke Afrikaans. Two (2) of the patients were married and eleven (11) had children. Twelve (12) were unemployed and five (5) were employed in low-paying jobs. See Table 1 for further details.

**Table 1:** Patient demographics

Patients Pseudonym	Sex	Age	Highest education completed	First Language	Marital Status	Children	Employed
Bianca	F	28	Grade 7	Afrikaans	Single	Yes (2)	No
Shane	M	29	Grade 12	Afrikaans	Single	No	No
Bongani	M	31	Grade 7	IsiXhosa	Single	No	Yes (Contractor)
Kaitlin	F	48	Grade 2	Afrikaans	Single	Yes (4)	No
Buhle	F	29	Grade 12	IsiXhosa	Married	No	No
Fezeka	F	49	Grade 7	IsiXhosa	Single	Yes (3)	Yes (Domestic Worker)
Aphiwe	M	26	Grade 10	IsiXhosa	Single	No	No
Lulama	F	41	Grade 12	IsiXhosa	Single	Yes (2)	Yes (Call Centre Attendant)
Nandipha	F	30	Grade 12	IsiXhosa	Single	Yes (1)	Yes (Baker)
Mncedisi	F	18	Grade 11	IsiXhosa	Single	Yes (1)	No
Ndiliswa	F	43	Grade 11	IsiXhosa	Married	Yes (4)	No
Yaseen	M	37	Grade 7	Afrikaans	Single	Yes (3)	No
Morne	M	50	Grade 6	Afrikaans	Single	No	No
Thandiwe	F	42	Grade 11	IsiXhosa	Single	Yes (1)	No
Zintle	F	29	Tertiary	IsiXhosa	Single	Yes (1)	No
Babalwa	F	29	Grade 11	IsiXhosa	Single	Yes (2)	Yes (Photographer)
Themba	M	29	Grade 11	IsiXhosa	Single	Yes (2)	No

The themes that emerged from the data are described below with sub-themes. These are: 1) lack of PCC, 2) patients’ understanding of the clinical space and knowledge of TB, and 3) patients’ expressed needs prior to discharge. Sub-themes within each category are summarised in Table 2. In the section that follows, findings from patients, health care workers, and policy makers are presented within each of these three main themes. There were overlapping responses among participants about many of the themes which emerged.

**Table 2:** Themes and sub themes

Themes	Sub themes
Lack of Patient-centred care	Inadequate TB education for newly-diagnosed patients and limited engagement to understand their needs and feelings
	Disease-focused approaches favoured over patient-focused approaches
	A sense of hopelessness and anxiety regarding TB treatment plans
Patients’ poor understanding of the clinical space and knowledge of TB	Reasons for hospitalisation in the context of a decentralised primary healthcare model and poor knowledge of TB prior to admission
Needs expressed by patients post-diagnosis and prior to discharge	No shared decision-making
	Patients’ family could support or thwart treatment adherence
	Socio-economic barriers to patients’ agency for CoC

### Lack of patient-centred care

#### Inadequate TB education for newly-diagnosed patients and limited engagement to understand their needs and feelings

Patients had no designated and consistent form of education about TB within hospital settings. In Hospital 1 “Matilda”, a professional nurse (PN), indicated that while they had training programmes for their staff, there was no such programme to teach *patients* about TB.

*Matilda (PN): We don’t always speak to the patients, but at times we will speak to the patients if they know about the results… We do not have a structured programme to train or educate the TB patients. We have a structured programme to train our staff.*

Similarly, in Hospital 2 medical doctor (MD) Dr. Christopher suggested that although there was a need for patient education, HCWs are limited by time and a heavy patient-load, particularly in larger hospitals. As a result, patients are not provided with sufficient information to look after themselves.

Joan, a policy maker in the WCDoH, provided an extensive narrative about the importance of improving patient knowledge, as emphasised in local policies and global recommendations. She felt that existing policies were not being adequately applied and patient education was not a priority, which she noted had not changed since her own postgraduate study two decades before. Policy makers such as Joan were frustrated by the gap between policy and practice. Although it is known that many patients are diagnosed at hospitals, they do not seem to offer any structured programmes patient TB education.

Apart from the limited or non-existent education received by patients at the hospital, patients expressed the need for a space in which they could discuss how they internalised the healing process. They wanted the space to articulate their own needs and feelings about their diagnosis and treatment at the hospital.

*Aphiwe (patient): They will not ask how you feel. There is nothing they are going to share with you, like lessons. There’s nothing. They are just treating here with medicine.*

Aphiwe was troubled about the TB medication given to him and specifically, he was upset that he was given medication without any engagement about his feelings about his stay and treatment at the hospital. Aphiwe seemed to want to know more about the disease but felt there was no opportunity to ask questions or obtain information from health workers.

Some health workers agreed with Aphiwe’s sentiment and explained that cultural and linguistic barriers played a role in their lack of engagement with some patients. In her interview, Dr. Bayley described the language issues that could further limit a deeper understanding of patients’ needs.

*Dr Bayley (MD): A lot of us don’t speak Xhosa (one of the local African languages), so I am trying to counsel somebody from a non-native language. It is difficult because my home language is English… Sometimes [important information and understanding] can get lost in translation because my Xhosa is not particularly good, their English is not particularly good, so there can be confusion…*

Dr. Bayley further explained that just because patients lived in an area where there is a high burden of TB, this did not mean everybody in the community had detailed knowledge about TB, as some might assume. She highlighted important cultural issues that had to be understood, based on more positive experiences at her previous workplace (a different) hospital, and felt that community-based counsellors could help bridge this gap.

It seemed in both categories of HCWs (nurses and doctors), there are different perceived reasons for a lack of effective TB education. From the nurses’ perspective, educating patients is not part of their routine practice of care. Doctors, on the other hand, pointed to language barriers as a key problem. It is likely that nurses, who spend more time with patients, might be able to engage more with patients if the counselling of TB patients was integrated into the structure of their standard care practices. However, this would not have addressed doctors’ concerns about language barriers between them and their patients.

#### Diseased-focused approaches favoured over patient-focused approaches

The above experiences by both staff and patients suggest that within hospital settings, understanding and responding to the patient as an individual with specific needs was not a priority. In light of the cultural issues described above, one policy maker thought that incorporating the cultural values of their patients could help make clinical and educational approaches more patient-focused. She was also concerned that oftentimes, health workers would make assumptions about a patient’s needs and level of understanding, rather than asking the patient directly.

*Joan (WCDoH): … the education should not be disease-focused. [We make many] assumptions… we [do not ask] the patient, ‘what do you know? What do you feel? What are your fears? What is your challenge?’ We are not asking, we are assuming we know it. So, we respond to our own assumptions…. we are not addressing the patients’ needs.*

Joan further worried that there was a heavier focus on patients’ medical condition and on reporting metrics than on the wellbeing and broader social and economic context of TB patients as they prepared to leave the hospital.

*Joan (WCDoH): Around the management of the TB patient, it’s almost as if the patient is not as important as me reporting the number of tablets that I have given out... Yeah, [but nothing in that patient’s life] has changed. [The patient] still does not have food security. And it is not the person alone. You must see that patient in the context of his family responsibilities. And if he doesn’t have food security, his family doesn’t have food security. And he [might] make the choice [to attend the clinics and take his medication], maybe not for him, but he would make the choice for his children.*

This over-emphasis on medication again exemplifies a disease- rather than patient-centred approach. Joan also recognised the importance of family and how patients might be worried about not only their medical condition but also, what they must do to look after their families’ broader needs.

#### A sense of hopelessness and anxiety regarding TB treatment plans

The information about a TB diagnosis was not always accompanied by a proper explanation of the implications that followed. In this study, patients were asked how they had felt when they heard that they had been diagnosed with TB, the kind of TB they had, and how they felt about taking treatment for TB. Their narratives reflected a strong sense of hopelessness and lack of autonomy.

*Bianca (patient): What can they do now about my stomach? All they can do is to give me my tablets. Yes, they cannot do anything now… I can’t just lie here. All the doctors have done everything for me. So, I can’t just lie here for nothing.*

Bianca was concerned about the state of her health and did not feel equipped or empowered to manage her own treatment, based on the information she had received. Bianca was asked if she thought her TB would get better if she finished the treatment given to her, but she did not think it would get better. Bianca did not respond when asked why she was not confident in the medication. Her body language and facial expressions paralleled her verbal expressions of hopelessness. She did not seem to have received any counselling, despite her distress. This lack of support is in contravention of proper PCC principles.

Similarly, Kaitlin explained to the first author that she was not planning to take (“drink”) all her medication and seemed generally overwhelmed, not only about the medication but also with concern about her living conditions. She had not had the same conversation with the nurse or the doctor.

*Kaitlin (patient): Sometimes I feel... [Kaitlin’s voice cracks as if she wants to cry] I have to take three [now], three [later]. I can’t drink all seven [pills], if I drink all [take all my pills at one time] I will die… It’s my skin – I can’t even buy lotion, look at it! I feel… I want to go home and sort out my children… I feel depressed.*

According to the tenets of PCC, patients receiving a diagnosis or medication should be asked how they feel afterwards, and should be provided with coping mechanisms.

Another participant, Ndiliswa, felt that her diagnosis was irresponsibly and incompletely provided to her, and only after she caught a nurse’s attention.

*Ndiliswa (patient): So, there was a sister who was working at night. They were sitting discussing... I was coming from the toilet and I said ‘hi’… So, the sisters came to me and said, ‘Did the doctor come and tell you what was going on with you?’ I said, ‘no, the doctor didn’t come. I was waiting for him to come and explain to me what is going on with me.’ The sister said, ‘The doctor told me that I must tell you that you have got MDR[-TB]’*

The fact that Ndiliswa was waiting for the doctor to provide her with the test results, but only received it through a nurse on duty also made her anxious about her medical condition. An MDR-TB diagnosis required a more focused and clear explanation to ease any form of anxiety that the patient might feel. Ndiliswa did not have the opportunity to receive such an engagement by health staff.

### Patients’ poor understanding of the clinical space and knowledge of TB

#### Patients’ reasons for hospitalisation in the context of a decentralised primary healthcare model and poor knowledge of TB prior to admission

The lapses in PCC described above were not the only problems that affected the care of patients diagnosed with TB at a hospital. Patients’ lack of understanding of the decentralised primary health care model in South Africa and poor knowledge about TB also created unmet expectations. Patients explained that when they had felt pain, or when an existing pain had worsened, they had gone to a hospital (rather than a clinic) to find relief. They had not seemed to recognise the symptoms of TB, or to know that they should first seek care at their primary facility. Two of the patients came to the hospital because they were concerned that they might die if they stayed at home.

*Bianca (patient): I was feeling pain in my stomach… I was feeling very sick.*

*Kaitlin (patient): Everything I eat is stopping here (pointing to her chest). I was asking what’s going on. I was becoming thin[ner] and thin[ner].*

Many of these patients could have been treated at a local clinic, but assumed that they should instead go to a hospital because their pain was very bad or unexplained, or because of the common local perception that they would receive better care at a hospital.

Patients also showed little or no knowledge about the aetiology and transmission pathways of TB. Although most had heard about TB prior to hospitalisation, the basics of decentralised care (and importance of CoC) were not well understood. Participants did not associate their pain and discomfort with TB. Instead, they came up with alternate explanations that showed a lack of TB knowledge and treatment literacy, even after they had been diagnosed and had initiated treatment (i.e. by the time they were interviewed). Patients expressed a lot of fear in relation to this lack of information. When asked if they knew anything about TB, patients responded in the following ways:

*Buhle (patient): I don’t know whether it is the treatment or it’s my body. I don’t know anything about TB.*

*Bongani (patient): I don’t know. I am confused. I know nothing about TB. It is the first time I have ever had TB. I always [said I didn’t] want to have TB. I was always afraid to have TB… I am afraid of TB.*

Even when patients had heard about different types of TB, knew it was dangerous or fatal, or were afraid to contract it, they displayed little or no knowledge of exactly how TB would affect them, and were unsure how to manage TB after discharge from hospital. The patients dwelled much more on their socio-economic needs and lack of support from hospital staff prior to discharge than on their health status and TB post-discharge.

### Needs expressed by patients post-diagnosis and prior to discharge

#### No shared decision-making

Patients were not generally consulted during the discharge planning phase. HCWs explained that their engagement with patients was only so as to provide a referral letter for continued treatment at their local clinic. When asked about their role in planning for their discharge, patients said that they were not consulted and were just “waiting for their discharge letter”.

*Buhle (patient): They have not told me anything …*

*Aphiwe: I am just listening to them. I do what I have to… they just give you the letter and tell you to take the letter to the local clinic so that they can know that you are coming from the hospital.*

An HCW, Matilda, agreed that the patients’ sole role in the discharge process was to collect their discharge letter and find the clinic that they had been referred to. Health workers were only expected to conduct the usual TB surveillance process (gathering relevant reporting metrics), sometimes without even talking to the patient, and to inform the patient about the location of their referral clinic.

*Matilda (PN): When we get results of positive TB, I go and see the patients. And specifically, check whether they are in the wards. Does the staff know? And then we complete the TB surveillance form to see where the patient has been admitted from, is there any co-morbidities, specifically HIV and what kind of TB is it… We don’t always speak to the patients… just to ensure that the patient has his discharge letter, his medication that he should get for five days and he/she should know exactly where to go… because there are… many clinics in [the local area].*

These HCW and patient responses resonated with what some policy makers had also highlighted: that not much attention is given to what patients had to say regarding their home situation, care needs, or fears about the process of discharge. Thus, patients receive instructions from HCWs but are not considered active partners in the discharge process.

#### Patients’ family situations could support or thwart treatment adherence

In light of their living conditions and low income, many patients were very anxious as they prepared to leave the hospital. Family involvement emerged as a crucial aspect of patients’ lives. When family relationships were strong, family was considered a supportive factor. However, when family relationships were difficult as was the case for some, it caused a lot of pain and worry about discharge.

*Kaitlin (patient): I want [my] older children to leave [home]… [crying]*… *Since I [have been in hospital], no one has come to visit me…. Last night I asked God to come take me. I feel so depressed, I feel so … I don’t know how to say it. I feel alone [still crying]. I can’t sleep in the night… [Another lady in my ward] is the one that supports me. She gives me anything I ask [coughing] because I help her [with her legs], I put her legs up and she gives me juice. I have no one in my life.*

Portia (PN), an HCW at Hospital 1, also emphasised the value of family involvement in supporting a patient’s treatment plans, particularly for poorer patients.

*Portia (PN): Most of [the TB patients] are thin, undernourished, so we refer them to the dietician, speak to the family, if the family comes and asks. The TB patients can all get [social welfare] grants while they are on treatment for six months. And most [of our]… TB patients are poor.*

As Portia highlighted, families are sometimes included in treatment discussions and this can facilitate adherence. However, she explained that this only happens if the family comes to the hospital themselves and asks about the treatment plan or about seeking government social assistance. For those who do not have supportive families, the treatment journey may be much more difficult, as expressed by Kaitlin, or may even be thwarted by family circumstances.

#### Socio-economic barriers to patient’s agency for CoC

The patients continued to refer to support – or lack thereof – at home as they prepared for discharge. Kaitlin’s immediate concern was not only her family but also her income situation. Continued clinic attendance and treatment adherence was the least of her worries. Not only was she not involved in discharge planning, which would have given her the opportunity to express her immediate needs and perhaps gain referrals to government or other support schemes, but she was also anxious about what would become of her financial status and family support after discharge from hospital. Bianca and Themba also expressed financial concerns that had already negatively affected their ability to adhere to treatment, or would in future.

*Kaitlin (patient): There are many things I need to make right in my house. I need to go home. I was so full of depression. It is too far from [home] to [the hospital]… [My family] don’t have money. They don’t even have money to buy food… my problems are too heavy. My children are not working.*

*Themba (patient): But I want [the government] to first see how I live before they give me something [apparently in reference to government social assistance cash transfers, to support him during his recovery]… I need support from government… If I can fix the way I [live] first, [government] can support me [with] money until this treatment [is finished]. After that I will say I can carry on and see. Because when I have power, I can be able to work.*

Themba believed that his “power” (strength) could be restored when his needs at home were met. He compared treatment with basic “support”, which is not necessarily money but rather secure access to housing and food. Themba acknowledged that the medication that he was expected to take after discharge was crucial to his recovery from TB, but he also believed that this should be complemented with support in other aspects of his life (particularly financially), so that he could be enabled to continue his treatment.

Health care workers expressed similar concerns about their patient’s needs beyond medication. For example, Alice (a nurse), highlighted the additional complication of high levels of substance abuse and addiction in the areas they serve. There are social workers in the hospitals whose main role is to make sure that patients have transportation home and are able to locate their family’s addresses and get subsequent help from families. In theory, these social workers would meet some of the support needs described above but they too felt overwhelmed with the scale of the socio-economic challenges that their clients faced. Linda, a social worker, described similar socio-economic problems (confirmed by patients and other HCWs) and specifically noted the cyclical relationship between TB and poverty. She highlighted that ironically, although people from a higher socio-economic class are less likely to get sick with TB, they have access to far better-resourced health services. Linda cited the importance of a full and detailed patient history for providing appropriate care and counselling, but said that diagnosing doctors generally do not have (or take) the time to take a history or to provide patient education about TB aetiology and transmission.

The socio-economic circumstances of TB patients featured significantly in both patient and HCW narratives as a key barrier to treatment adherence. It was suggested that a detailed patient history could reveal expected difficulties with the continuation of a patient’s care post-discharge, and perhaps indicate where specific pre-discharge care or support could be provided to moderate these potential barriers.

## Discussion

This paper contributes to our understanding of patients’ experiences and perceptions of treatment of TB before discharge from a tertiary or district hospital in the Western Cape province of South Africa. Based on a model drawn from the concept of PCC, we described lapses in the pre-discharge care of patients diagnosed with TB in two Western Cape hospitals. Although there are other aspects of PCC [12], the three categories described above are used in this article as a framework for understanding how the hospitals where patients were diagnosed with TB performed in achieving PCC (See Fig. 1). Our findings describe a range of challenges TB patients confront in these TB wards, most notably in the form of insufficient information, a disease-focused (rather than patient-focused) approach to care, lack of shared decision making, and the complexities of family and socioeconomic contexts. These challenges often work against patients’ best efforts to remain adherent on TB treatment after they leave the hospital.

A key thread running through all of these findings is an underlying lack of empowered patient involvement in understanding their disease and managing their treatment journey. This lack of empowerment for patients was perhaps most evident in the poor education many TB patients reported receiving about their TB diagnosis and the treatment regimen they were initiating. Thorough patient education has been identified as critical to patients’ motivation and ability to follow through with TB care after discharge [3, 9, 10, 21]. Education for all patients with TB is important but it is particularly important [3, 21] to provide extra attention and specialised care and education for patients diagnosed with DR-TB (a notable gap in this study).

There were a number of forces underlying this lack of basic patient education and engagement. The management of TB in these two hospitals was strongly disease focused, an approach characterised by a heavy emphasis on medical intervention and relatively little attention to patients’ needs and experiences. This kind of medicalised approach can represent a significant threat to the success of a patient’s care [22–25]. This approach is in turn reinforced in settings where there is inadequate human resources [9, 26]. With limited knowledge of TB prior to admission and a lack of PCC in the hospitals, it is unlikely that patients will develop a clear understanding of the importance of continued care and adherence after discharge [9, 12, 23].

When there were efforts to provide health education, patients faced a number of critical barriers to communications. Lack of effective clinical communication is a pervasive problem in the South African public sector health system [26, 27]. Communication problems are in part about linguistic challenges between HCWs and patients, which can lead to a patient’s inability to understand what is being said or a health worker’s inability to express what they mean, especially in an extremely multilingual context (South Africa has 11 official languages and many sub-dialects) [28]. Even when there is a shared language, though, health education can be seriously inadequate [26] leaving patients unclear on the implications of their diagnosis and the impending treatment journey. Finally, entrenched hierarchies between patient and provider and extremely high workloads among providers can leave many patients feeling incapable of asking HCWs for clarification or further information [27]. The impact of poor communication were clearly evident in this study, with many participants expressing inadequate treatment literacy and an inability to ask questions. Spending more time speaking with patients in a patient-centred manner so that they properly understand their diagnosis treatment is critical to allaying fears and anxieties, and contributing to the continuity of patients’ TB care [21, 27, 29].

The patients in this study faced an enormous array of challenging family, community, and socioeconomic hurdles to their adherence, and they are the ones who are in the best position to not only express these “complexities” and “life issues”, but also to be able to contribute to discussions of how to negotiate these and embark on their personal treatment journey [30]. For most of them, however, their relationship to healthcare providers was characterised by powerlessness. Within the framework of PCC, patients were not considered active partners in decision-making in the hospital about the issues that would directly affect their health and by extension, the health of the population. These patients experienced a double layer of powerlessness with their agency challenged by both the one-sided decision-making in the health system as well as their own lack of agency in the face of oppressive structural conditions like material insecurity and family dynamics, which are documented by many authors within and outside South Africa [15, 31, 32].

HCWs seemed well-aware of the many socio-economic barriers patients faced, but in their responses, they often attributed patients’ inability to overcome these barriers to a lack of agency. Instead of acknowledging the challenge represented by these barriers and working with patients to find ways of remaining on TB treatment in spite of these challenges, the main concern for HCWs was for patients to get access to their medication and to move on to the next level of care. It is crucial to always recognise, though, that the impact of responding to so many sick people with so few staff takes its toll on the HCWs in the public sector health system [33, 34]. Staff shortages continue to be an ongoing problem in South Africa’s health sector. Placing lay counsellors tasked with TB education at hospitals, as suggested by one HCW participant, may be a useful step toward improving access to information. It may also be helpful to reemphasise the importance of educating patients by embedding this in the daily service protocols of healthcare staff in hospitals. In addition, it may be helpful if there is transformation of the education programme with more emphasis on language learning in medical and health professions training. Counsellors could be encouraged to be enrolled in this training programme because it might be difficult for medical staff to be involved in this due to their already overwhelming clinical workload. Cultural issues go beyond language but the ability for the health workforce to communicate in the local first language would also facilitate improved communication.

Finally, in terms of clinical implications, improvements in PCC for TB patients may be especially useful in minimising rifampicin-resistant TB [35]. TB patients have complex and multi-faceted needs and challenges that include significant socio-economic and medical problems and require a nuanced approach [35]. Some studies have demonstrated that improvements in patient-health provider relationships can enhance health outcomes in patients with a wide variety of health problems [36, 37]. This may include developing a trusting relationship between providers and recipients of TB care, which can be part of the TB DOTS plan in the South African health system [38].

## Limitations

One limitation of this paper is that it only reports on the perceptions and experiences of these patients prior to discharge. A forthcoming paper will detail the post-discharge experiences of patients in their referral clinics, which should be a useful complement to this paper by further expanding our understanding of PCC and its impact on TB CoC in the South African health system. As it was not possible to move patients out of their ward, some patients were interviewed in the same ward. Some patients might not have felt entirely relaxed being interviewed while other people were present, but they were all interviewed individually and with as much privacy as possible. The first author previously worked in one of the hospitals. Navigating the clinical space was much easier because of a close association with some former work colleagues. However, this association was only with one of the two hospitals, and reported patient and healthcare experiences were similar across both settings. To minimise any potential bias from two participants who knew the first author, all participants were encouraged to be honest in their answers as these would help inform a better healthcare provision for patients infected with TB. Even though the two patients diagnosed with DR-TB expressed similar experiences with TB patients, it is likely that more patients from this group could have provided more insights on the experiences of patients with DR-TB. However, these two were the only newly-diagnosed patients during the time of recruitment of patients. Finally, the data were collected between 2015 and 2017, so it is possible there have been some changes in inpatient clinical management and discharge planning processes since then.

## Conclusion

Improving the three intersecting elements of (TB education, engaging with patients’ needs and feelings, and shared decision-making) has the potential to positively influence patients’ CoC, which should have a positive impact on TB recovery and incidence rates, as well as limiting the creation of new drug-resistant strains. This article has described the ways in which the hospitals where this study was conducted did not achieve PCC, as mandated by South African health system guidelines and governing documents. PCC is a key component in patients’ CoC as it makes the patient an active participant in their treatment journey, which recognises patient values and needs [39]. The hopelessness, powerlessness and lack of partnership that emerged when the patients accessed medical care in the hospitals were the result of a range of problems including HCWs’ time constraints and heavy workloads, which detracted from a focus on patients’ needs and feelings. Key recommendations in the paper include the introduction of a cadre of adequately trained TB counsellors in the hospitals who could provide more detailed education and information, and might help to navigate cultural and linguistic challenges between HCWs and patients. This may enhance PCC in hospitals before discharge, which can contribute to patients’ continuity of TB care upon discharge from hospitals.

## Supplementary Information

**Additional file 1.** Analytical steps.

**Additional file 2.** Checklist, COREQ (Consolidated criteria for Reporting of Qualitative research).

## Data Availability

The data are uploaded to the University of Cape Town’s data repository but are not publicly available because of potentially identifiable and sensitive information. They are available upon request to the administrator of the UCT data repository, Dr. Sanjin Muftic, at dls@uct.ac.za.
