# Supplementary material for: A qualitative study of patients and healthcare workers’ experiences and perceptions to inform a better understanding of gaps in care for pre-discharged tuberculosis patients in Cape Town, South Africa
Source: BMC Health Serv Res. 2022 Jan 29;22:128. doi: 10.1186/s12913-022-07540-2 (PMC8801106; doi:10.1186/s12913-022-07540-2)
Supplement: Supplementary file 1 — Additional file 1. Analytical steps. [file 12913_2022_7540_MOESM1_ESM.pdf]

Steps of analytical method

Data reduction (Descriptive codes)

The process of reading, editing and segmenting the data. The point of also assigning labels to attach meaning to the pieces of data and these labels serve several functions: they index data, provide a basis for storage and retrieval and further analysis.

Reviewer 1

Patients	Healthcare workers	Policy makers
Reasons for hospitalisation	Experiences of inpatient care	Knowledge of inpatient care
Knowledge of TB	Experiences of patient education	Knowledge of patient education
Knowledge of discharge process	Description of roles	Knowledge of discharge process
Experiences of inpatient care	Knowledge of linkage between tertiary/district and primary care	Knowledge of other kinds patient support
Experiences of home circumstances	Perceptions of influences of attendance to primary care	Perceptions of attendance at clinics upon discharge
Perceptions of continuity of care		Understanding of the link between tertiary and clinic

Data display

The process of organising, and assembling information because qualitative data is typically voluminous, bulky and dispersed. This connects with data reduction, but specifically it is a display of how the reorganising of data has been done to provide a clear picture as to which stage the analysis has reached and are the basis for further analysis. Linking of key responses between participants

Reviewer 1

<table>
<tr><th>Participants</th><th>Similar key responses</th></tr>
<tr><td>Patients</td><td rowspan="3">

No structured programme for education of patients
Patients not adequately counselled

Why?

Time constraints
Huge burden of patients
Cultural and linguistic barriers
Different focus – treatment and discharge rather than spending time with patients

Implications?

Limited understanding of TB prior to discharge
Anxiety and uncertainty

</td></tr>
<tr><td>Healthcare workers</td></tr>
<tr><td>Policy makers</td></tr>
</table>

Patients	• Limited knowledge of TB<br>Why?<br>• Bypass primary care - where primary TB education happens?<br>• Partly failures of decentralised system, partly cultural?<br>• Not a partner in discharge process<br>Why?<br>• Similar themes expressed by all participants above<br>Implications?<br>• Feelings of hopelessness, powerlessness<br>• Patients not in charge of treatment journey

Verifying conclusions

A final process of justifying the reasons for reducing and displaying data, which will assist in drawing conclusions. These conclusions are in the form of propositions and themes. Other research team members verify these conclusions to enhance inter-coder reliability.

Reviewer 1,2 & 3

**Key thematic areas**	**Sub themes**
Lack of patient-centred care	Inadequate TB education for newly-diagnosed patients
	Disease-focused approaches favoured over patient-focused approaches
	A sense of hopelessness
	Limited engagement with patients so as to understand and respond to their needs and feelings
Patients' understanding of the clinical space and knowledge of TB	Reasons for hospitalisation in the context of a decentralised primary healthcare model
	Poor knowledge of TB among patients prior to admission
Patients' expressed needs prior to discharge	No shared decision-making
	Patients' complex family situations
	Socio-economic barriers to patients' agency for CoC

- Combining sub-themes for clearer presentation in the manuscript
  - Reasons for hospitalisation and poor TB knowledge are related
- Reorganise sub-themes for clarity in the manuscript
- Presented findings based on the patient-centred care model used in the study
